# Do educated women in Sierra Leone support discontinuation of female genital mutilation/cutting? Evidence from the 2013 Demographic and Health Survey

**DOI:** 10.1186/s12978-020-01027-1

**Published:** 2020-11-07

**Authors:** Edward Kwabena Ameyaw, Sanni Yaya, Abdul-Aziz Seidu, Bright Opoku Ahinkorah, Linus Baatiema, Carolyne Njue

**Affiliations:** 1 School of Public Health, Faculty of Health, University of Technology Sydney, Sydney, NSW Australia; 2 School of International Development and Global Studies, University of Ottawa, Ottawa, Canada; 3 The George Institute for Global Health, The University of Oxford, Oxford, UK; 4 Department of Population and Health, College of Humanities and Legal Studies, University of Cape Coast, Cape Coast, Ghana; 5 College of Public Health, Medical and Veterinary Sciences, James Cook University, Townsville, QLD Australia

**Keywords:** Discontinue, Female genital mutilation/cutting, Reproductive health, Sierra Leone, Women

## Abstract

**Introduction:**

Female genital mutilation/cutting (FGM/C) comprises all procedures that involve the total or partial elimination of the external genitalia or any injury to the female genital organ for non-medical purposes. More than 200 million females have undergone the procedure globally, with a prevalence of 89.6% in Sierra Leone. Education is acknowledged as a fundamental strategy to end FGM/C. This study aims to assess women's educational attainment and how this impacts their views on whether FGM/C should be discontinued in Sierra Leone.

**Methods:**

We used data from the 2013 Sierra Leone Demographic and Health Survey. A total of 15,228 women were included in the study. We carried out a descriptive analysis, followed by Binary Logistic Regression analyses. We presented the results of the Binary Logistic Regression as Crude Odds Ratios (COR) and Adjusted Odds Ratios (AOR) with 95% confidence intervals (CIs).

**Results:**

Most of the women with formal education (65.5%) and 15.6% of those without formal education indicated that FGM/C should be discontinued. Similarly, 35% of those aged 15–19 indicated that FGM/C should be discontinued. Women with a higher education level had a higher likelihood of reporting that FGM/C should be discontinued [AOR 4.02; CI 3.00–5.41]. Christian women [AOR 1.72; CI 1.44–2.04], those who reported that FGM/C is not required by religion [AOR 8.68; CI 7.29–10.34], wealthier women [AOR 1.37; CI 1.03–1.83] and those residing in the western part of Sierra Leone [AOR 1.61; CI 1.16–2.23] were more likely to state that FGM/C should be discontinued. In contrast, women in union [AOR 0.75; CI 0.62–0.91], circumcised women [AOR 0.41; CI 0.33–0.52], residents of the northern region [AOR 0.63; CI 0.46–0.85] and women aged 45–49 [AOR 0.66; CI 0.48–0.89] were less likely to report that FGM/C should be discontinued in Sierra Leone.

**Conclusion:**

This study supports the argument that education is crucial to end FGM/C. Age, religion and religious support for FGM/C, marital status, wealth status, region, place of residence, mothers' experience of FGM/C and having a daughter at home are key influences on the discontinuation of FGM/C in Sierra Leone. The study demonstrates the need to pay critical attention to uneducated women, older women and women who have been circumcised to help Sierra Leone end FGM/C and increase its prospects of achieving Sustainable Development Goals (SDG) three and five.

## Plain English summary

Sierra Leone has one of the highest rates of FGM/C in the world. It is the only country in south western Africa with a very high FGM/C rate. The most recent Demographic and Health Survey (DHS) reported a national prevalence of 89.6%. In this study, we investigated women's educational attainment and their beliefs on whether FGM/C should be discontinued in Sierra Leone. We conducted descriptive analysis followed by binary logistic regression analyses. Our results were presented as crude odds ratios (COR) and adjusted odds ratios (AOR) with 95% confidence intervals (CIs), signifying the level of precision. We noted that women who had a higher education level were more likely to report that FGM/C should be discontinued [AOR 4.02; CI 3.00–5.41]. Christian women [AOR 1.72; CI 1.44–2.04], women who said that FGM/C is not required by religion [AOR 8.68; CI 7.29–10.34] and wealthier women [AOR 1.37; CI 1.03–1.83] were more likely to indicate that FGM/C should be discontinued. In contrast, women in union [AOR 0.75; CI 0.62–0.91] and circumcised women [AOR 0.41; CI 0.33–0.52] were less likely to support the discontinuation of FGM/C in Sierra Leone. Our study supports the argument that education is crucial for ending FGM/C. The study illustrates the need to pay particular attention to uneducated women, older women and women who have been circumcised to help Sierra Leone end FGM/C and increase its prospects of achieving SDG three and five.

## Introduction

Female genital mutilation (FGM), also referred to as female genital cutting or circumcision (FGC), comprises "all procedures that involve partial or total removal of the external female genitalia, or another injury to the female genital organs for non-medical reasons" [1]. There are four main types of FGM/C. Type 1 constitutes either a total or partial removal of clitoral glans and/or clitoral hood, while type 2 involves the total or partial removal of the clitoral glans and inner folds of the vulva. The third type—also called infibulation—is characterised by the narrowing of the vaginal opening, while type 4 constitutes any other harmful procedures to females' genitalia for non-medical reasons [1]. More than 200 million females globally have undergone the procedure [1]. People at risk include young girls between infancy and adolescence and in some instances, adult women. Globally, more than three million girls are estimated to be at risk annually [1]. FGM/C has been documented in about 30 countries in Africa, the Middle East and Asia [1, 2]. Currently, FGM/C abandonment interventions trail behind the incidence rate and its prevalence is anticipated to escalate within the next decade if efforts are not expedited [3].

Traditional circumcisers usually execute FGM/C; however, due to adverse health concerns, healthcare providers are involved at times in countries like Egypt, Kenya, Indonesia and Malaysia [2, 4]. The WHO has therefore adopted strategies to deter health workers from the practice [5]. FGM/C subjects women to cruelty, torture and inhuman treatment, and can lead to the contraction of infections including HIV and AIDS [6–8] and even death [1]. It is associated with various short- and long-term health implications, depending on the type. Some of the short-term consequences are haemorrhage, repetitive infections of the low urinary tract and acute anaemia [9–11]. The long-term consequences include pregnancy and childbirth complications and infertility [1, 12, 13]. Multiple psychological implications have also been reported, including low self-esteem, psychiatric diagnoses, anxiety, somatisation and phobia [14, 15].

In many communities, FGM/C is performed on very young females under the age of 18 with devastating results. A report from Sierra Leone in 2018, for instance, revealed that a 10-year-old girl died after undergoing FGM/C as part of a mass initiation of 68 girls into a secret society [16]. The ages of the girls who participated in this initiation ranged from nine to 12 [16].

These and several other appalling implications of FGM/C have led to a strong global call to eliminate FGM/C. A considerable number of UN human rights treaty monitoring bodies such as the Committee on the Elimination of All Forms of Discrimination against Women, the Committee on the Rights of the Child and the Human Rights Committee have condemned the practices and provided recommendations to help states end FGM/C [17]. Target 5.3 of the SDG emphasises the elimination of all harmful practices, such as child, early and forced marriage and FGM/C by 2030 [18].

The World Health Organisation (WHO), the United Nations Population Fund (UNFPA), other local and international human rights organisations and several countries have also introduced measures to end FGM/C. These include the Joint Programme on Female Genital Mutilation, implemented in 15 African countries [19]. The World Health Assembly (WHA) has also passed Resolution WHA61.16 resolution [20], which adopts a multi-sectoral approach to deal with FGM/C. Prevention and protection measures have also been taken globally to stop healthcare providers from performing FGM/C [5, 21]. For instance, there is the development of healthcare providers' tools and training to manage FGM/C-related complications and prevent new FGM/C cases [21].

FGM/C is one of the activities conducted during a rite of passage ceremony to transition girls to adulthood in Sierra Leone [22]. An influential women's group known as the Bondo society conducts the ceremony. The Bondo society is a secretive custom or tradition of some women in Sierra Leone, which is rooted in mythology and has FGM/C as its mainstay [23, 24]. Some women believe even talking about the society puts them at the risk of demons or curses [24]. During the ceremony, a traditional leader, locally called a Sowei, performs the act [23, 25].

Sierra Leone is a country with one of the highest FGM/C prevalence globally [26]. It is the only country in south western Africa with very high FGM/C rates [27]. To date, there is no explicit law that prohibits FGM/C in Sierra Leone [28]. There is no strong political will to combat against FGM/C in Sierra Leone, as the traditional FGM/C practitioners, locally known as Soweis, influence females’ votes and serve as the link between some communities and the central government [23, 29]. The practice is considered a social norm. Females who refuse it are sometimes ostracised and labelled as ill-mannered or unready for marriage and forfeit the public recognition earned by those who comply [30].

The most recent Demographic and Health Survey (DHS) of Sierra Leone reported a national prevalence of 89.6% [31]. Studies have been conducted on various dimensions of FGM/C in Sierra Leone and other sub-Saharan African countries to understand the possible drivers of the act [23, 32, 33]. Some evidence from Sierra Leone suggests that women were the main decision-makers of FGM/C and that educational attainment influences decision-making [34].

Factors associated with FGM/C discontinuation include the level of education, religion, ethnicity, urban residence, age at marriage [35], geographical factors [36] and male involvement [37]. Notably, there is a plethora of evidence that education can be an antidote to FGM/C by enlightening women about the practice’s shortcomings [38–41]. Overall, daughters of highly educated mothers are less likely to experience FGM/C, relative to daughters of mothers with little or no education [42]. However, it is worth noting that female education has generally been low in Sierra Leone compared to male education [43, 44]. There is a lack of evidence about the intersection between educational levels and FGM/C in Sierra Leone. A recent study explored the relationship between education and women’s intention to circumcise their daughters and reported an inverse relationship between education and FGM/C intention [45].

Since FGM/C is socio-culturally entrenched without any political commitment to abandon it [23, 29, 30], it is worth researching whether double standards exist regarding the abandonment of FGM/C. In Egypt, for instance, although educated women supported the discontinuation of FGM/C, those who had positive cultural conceptions of FGM/C were less likely to discontinue, regardless of educational attainment [46]. This study appears to be the first research that explores the educational attainment of women and women’s viewpoints on whether FGM/C should be continued in Sierra Leone. The study could benefit Sierra Leone’s public health position by highlighting the need for anti-FGM/C socio-educational Information, Education, and Communication/Behaviour Change Communication (IEC/BCC) interventions. It will be challenging to end FGM/C without identifying and appreciating women’s perceptions of the procedure, and how their education levels impact their views.

## Methods

### Study setting

This study was conducted in Sierra Leone, which shares boundaries with Liberia to the southeast and Guinea to the northeast. Sierra Leone has a tropical climate with a diverse environment, ranging from savanna to rainforests, representing a total area of 71,740 km^2^. The country's population according to the 2015 population and housing census was 7,092,113 [47]. More than half (4,187,016) of the population live in rural areas, whereas 41% (2,905,097) live in urban centres. Between 2004 and 2015, the country recorded an annual population growth rate of 3.2%. The sex ratio stands at 96.8 males per 100 females. The report also shows out of the 6,589,838 people aged three years and above, 55.4% have attended school while 44.2% have never attended school. The percentages of males currently in school (39.1%) and those who ever attended school (60%) are higher than their female counterparts (35.3% and 50.9% respectively). The proportion of the population that has never attended school in rural areas is 32.7% and 11.5% in urban areas. Freetown is the country’s capital and largest city. Agriculture is the dominant economic activity. About 78% of the population is Muslim and 21% is Christian. The country is divided into five administrative regions (Eastern, Northern, North West, Southern and Western Area) which are further divided into 14 districts [47].

### Data source

This present study utilised data from the women’s recode file of the 2013 Sierra Leone Demographic and Health Survey (SLDHS) [31]. This was the second Demographic and Health Survey (DHS) in Sierra Leone. DHS collates data to monitor the population and public health situation of surveyed countries. The 2013 SLDHS explored information on FGM/C and several maternal and child health indicators such as nutrition, exclusive breastfeeding and fertility. The Measure DHS determined the sample to derive a reliable estimate for important variables in the country. This occurred by considering the rural/urban settings together with the four administrative regions and all 14 districts. The sample was stratified and determined in two main stages to achieve representativeness [31]. The response rate for the survey was 99%. The sampling procedure is extensively documented in the report [31]. The authors were granted access to utilise the survey dataset from the Measure DHS Program's website. The dataset is available to the public via https://dhsprogram.com/data/available-datasets.cfm.

## Study variables

### Dependent variable

The dependent variable for this study was ‘discontinuation of FGM/C’, drawn from the survey question: ‘Do you think that this practice should be continued, or stopped?’ The question had the following responses: ‘continued—1’, ‘discontinued—2’, ‘depends—3’, and ‘don’t know—4’. All women who participated in the 2013 SLDHS responded to this question regardless of their FGM/C status. Our study included those who answered ‘continued’ coded as ‘0’ and ‘discontinued’ coded as ‘1’. We excluded those who answered ‘depends—3’ and ‘don’t know—4’, who comprised 4.6% and 3.1% of the total sample respectively. They were excluded to align the data to our study purpose (to investigate the respondents’ definite viewpoint on whether FGM/C should be discontinued). Out of the 16,658 women who participated in the SLDHS, 15,228 were included in our study.

### Independent variable

The principal independent variable for the study was ‘level of education’. The study followed the original categorisation of the variable by the DHS: no education, primary, secondary and higher. We adopted the ‘level of education’ as the principal independent variable because consistent evidence has suggested that education is associated with FGM/C continuation [45, 48–53]. Therefore, we wanted to explore women's position about the discontinuation of FGM/C, based on their educational level. Informed by previous studies on FGM/C [33, 41, 54–56], the researchers included some socio-demographic characteristics as covariates. These are age, religion, whether FGM/C is required by religion, wealth, marital status, respondent has daughters at home, residential status, social acceptance of FGM/C circumcision status and region. Religion was recorded as ‘Christianity—1’, ‘Islam—2’ and ‘Other—3’ to make it conceptually relevant and meaningful for analysis. In the DHS, wealth is a composite measure computed by combining data on a household’s ownership of carefully identified assets, including a television, bicycle, materials used for house construction, sanitation facilities and type of water access. Principal component analysis was used to transform these variables into a wealth index by placing individual households on a continuous measure of relative wealth. The DHS segregates households into five wealth quintiles: poorest, poorer, middle, richer and richest [57].

### Data analysis

The study employed a descriptive analysis followed by binary logistic regression analysis. The descriptive analysis calculated the women's proportion according to educational level, socio-demographic characteristics and the continuity/discontinuity of FGM/C, as shown in Table 1. The socio-demographic characteristics are presented as frequencies and percentages, and sample weight was applied at this stage to offset possible weaknesses such as unequal probabilities and non-coverage of some populations [58]. Significant associations were tested between education, each socio-demographic variable and the continuation/discontinuation of FGM/C. Significance was set at a 95% confidence level. Two binary logistic regression models were developed in a hierarchical manner. The crude model (Model I) only accounted for the level of education and continuation/discontinuation of FGM/C. The adjusted model (Model II), included all socio-demographic variables in addition to education (shown in Table 2).

**Table 1:** Education, socio-demographic characteristics and FGM/C (n = 15,228)

Variable	Weighted (n)	Percentage (%)	FGM		X^2^, p-value
			Continued (%)	Discontinued (%)	
*Education*					
No education	8597	56.5	84.4	15.6	172.3, p < 0.001
Primary	2116	13.9	77.0	23.0	
Secondary	4067	26.7	58.7	41.3	
Higher	448	2.9	34.5	65.5	
*Age*					424.4, p < 0.001
15–19	3426	22.5	65.0	35.0	
20–24	2446	16.1	69.4	30.6	
25–29	2603	17.1	75.0	25.0	
30–34	2123	13.9	79.7	20.3	
35–39	2097	13.8	81.1	18.9	
40–44	1275	8.4	83.5	16.5	
45–49	1258	8.2	85.8	14.2	
*Religion*					858.1, p < 0.001
Islam	11,997	78.8	80.3	19.7	
Christianity	3185	20.9	55.5	44.5	
Other	37	0.2	82.1	17.9	
None	9	0.1	100	0.0	
*FGM required by religion*					271.6, p < 0.001
Yes	8917	58.6	90.6	9.4	
No	5051	33.2	47.8	52.2	
Don’t know	1260	8.2	69.6	30.4	
*Wealth*					875.3, p < 0.001
Poorest	2875	18.9	85.1	14.9	
Poorer	2767	18.2	82.3	17.7	
Middle	2895	19.0	81.2	18.8	
Richer	3063	20.1	73.8	26.2	
Richest	3628	23.8	57.1	42.9	
*Marital status*					786.4, p < 0.001
Never in union	4212	27.7	59.3	40.7	
Currently in union	10,085	66.2	81.2	18.8	
Formerly in union	931	6.1	80.1	19.9	
*Residential status*					542.6, p < 0.001
Urban	5387	35.4	64.9	35.1	
Rural	9841	64.6	81.6	18.4	
*Daughters at home*					422.8, p < 0.001
0	7930	52.1	68.3	31.7	
1	4269	28.0	80.0	20.0	
2	2097	13.8	84.5	15.5	
3	735	4.8	87.0	13.0	
4	159	1.0	89.0	11.0	
5 or more	38	0.3	84.2	15.8	
*Circumcised*					201.9, p < 0.001
No	1467	9.6	39.8	60.2	
Yes	13,761	90.4	78.6	21.4	
*Social acceptance of FGM/C*					301.2, p < 0.001
No	7018	46.1	58.0	42.0	
Yes	8210	53.9	89.1	10.9	
*Region*					807.6, p < 0.001
Eastern	3388	22.3	78.4	21.6	
Northern	5774	37.9	79.3	20.7	
Southern	3132	20.6	79.9	20.1	
Western	2934	19.3	52.1	47.9	

*Source*: 2013 SLDHS

**Table 2:** Binary logistic results on education and FGM/C discontinuation

Variable	Model I		Model II	
	COR	95% CI	AOR	95% CI
*Education*				
No education	1	[1]	1	[1]
Primary	1.72***	[1.44–2.05]	1.28**	[1.07–1.54]
Secondary	4.46***	[3.78–5.26]	2.27***	[1.89–2.72]
Higher	13.24***	[9.58–18.30]	4.02***	[3.00–5.41]
*Age*				
15–19			1	[1]
20–24			1.20	[0.98–1.45]
25–29			1.19	[0.94–1.51]
30–34			1.04	[0.81–1.33]
35–39			0.92	[0.71–1.18]
40–44			0.71*	[0.54–0.93]
45–49			0.66**	[0.48–0.89]
*Religion*				
Islam			1	[1]
Christianity			1.72***	[1.44–2.04]
Other			0.78	[0.33–1.83]
None			0.51	[0.45–1.23]
*FGM required by religion*				
Yes			1	[1]
No			8.68***	[7.29–10.34]
Don’t know			3.94***	[3.13–4.96]
*Wealth*				
Poorest			1	[1]
Poorer			1.28*	[1.02–1.61]
Middle			1.16	[0.89–1.51]
Richer			1.37*	[1.03–1.83]
Richest			1.38	[0.92–2.07]
*Marital status*				
Never in union			1	[1]
Currently in union			0.75**	[0.62–0.91]
Formerly in union			0.75	[0.55–1.02]
*Residential status*				
Urban			1	[1]
Rural			1.09	[0.81–1.47]
*Daughters at home*				
0			1	[1]
1			0.82*	[0.70–0.95]
2			0.71*	[0.57–0.87]
3			0.50***	[0.34–0.73]
4			0.56	[0.29–1.09]
5 or more			1.28	[0.51–3.21]
*Circumcised*				
No			1	[1]
Yes			0.41***	[0.33–0.52]
*Social acceptance of FGM/C*				
No			1	[1]
Yes			0.26***	[0.22–0.30]
*Region*				
Eastern			1	[1]
Northern			1.11	[0.85–1.46]
Southern			0.63**	[0.46–0.85]
Western			1.61**	[1.16–2.23]

*Source*: 2013 SLDHS

*COR* crude odds ratio, *AOR* adjusted odds ratio

*p < 0.05, **p < 0.01, ***p < 0.001

All the socio-demographic variables were included because they were significantly associated with FGM/C continuation/discontinuation, as illustrated in Table 1. The survey command (svy) in STATA was applied to account for the survey's complex sampling procedure. Variance inflation factor (VIF) was used to investigate whether multicollinearity existed between the variables. It was found that there was no multicollinearity (maximum VIF = 3.8, Minimum VIF = 1.2, Mean VIF = 1.76). All analyses were conducted in STATA version 13.0.

### Ethics approval

Ethical approval for the 2013 SLDHS was provided by the Ministry of Health and Sanitation of Sierra Leone and the DHS Program's ethics committee. Informed consent was sought from all participants during the fieldwork. The authors applied and were granted access to utilise the data from the Measure DHS Program's website. The dataset is freely available to the public via https://dhsprogram.com/data/available-datasets.cfm.

## Results

### Education, sociodemographic characteristics, and discontinuation of FGM/C

More than half of the participants had no formal education (56.5%). In addition to education, all socio-demographic characteristics had significant associations with FGM/C's discontinuation. Most of the women who had formal education (65.5%) and 15.6% of those without formal education indicated that FGM/C should be discontinued. A significant number of all women surveyed were aged between 15 and 19 years (22.5%). 35% of those aged 15–19 indicated that FGM/C should be discontinued. Approximately 79% of the study participants reported their religion as Islam. About 24% were categorised as ‘richest’, and among these, 42.9% reported that the practice should be discontinued. Also, 66% of all women in the study were in a union; 18.8% thought FGM/C should be discontinued compared to 40.7% of the women who had never been in a union, who thought the practice should be discontinued. Most women in the sample resided in rural locations (64.6%). Among those not circumcised, six out of ten (60.2%) stated that FGM/C should be discontinued. More than half of the women (53.9%) reported that FGM/C is socially accepted, and of these, only 10.9% thought that the practice should be discontinued (Table 1).

### Binary logistic results on education and FGM/C discontinuation

Results from the binary logistic regression are reported in Table 2. In the unadjusted model (Model I), women with higher education were 13 times more likely to say that FGM/C should be discontinued, compared to those without education [COR 13.24; CI 9.58–18.30]. A similar observation was made when we included women’s socio-demographic characteristics, as shown in the adjusted model (Model II). Women with higher education had a higher likelihood of reporting that FGM/C should be discontinued [AOR 4.02; CI 3.00–5.41]. Women aged 45–49 were less likely to report that FGM/C should be discontinued [AOR 0.66; CI 0.48–0.89]. Christian women [AOR 1.72; CI 1.44–2.04], women who said that FGM/C is not required by religion [AOR 8.68; CI 7.29–10.34], wealthier women [AOR 1.37; CI 1.03–1.83] and women from the western part of Sierra Leone [AOR 1.61; CI 1.16–2.23] most commonly stated that FGM/C should be discontinued. However, women in unions [AOR 0.75; CI 0.62–0.91], circumcised women [AOR 0.41; CI 0.33–0.52] and those from the northern region [AOR 0.63; CI 0.46–0.85] were less likely to report that FGM/C should be discontinued (Table 2).

## Discussion

Using a nationally representative sample drawn from the 2013 DHS, this study investigated how educational levels of women of reproductive age in Sierra Leone influence their thoughts about continuity or discontinuity of FGM/C in their country. We found that women with a higher level of education were more likely to support the discontinuation of FGM/C than those without formal education. The impact of formal education efforts to end FGM/C have been significant [49]. A previous study from Sierra Leone also reported that as a woman’s educational attainment increases, her intention to subject her daughter to FGM/C reduces [45]. These findings potentially indicate that educated women in Sierra Leone do not support FGM/C continuation. This suggests that women’s education should be prioritised in Sierra Leone to end FGM/C and thereby enhance the country’s chances of achieving SDG target 5.3 [56]. Robertson (2013) explains that educated women are knowledgeable about the health implications of FGM/C and understand the short- and long-term implications of the practice [48]. It is vital to situate FGM/C in the context of intercultural education at basic and secondary levels of education to facilitate efforts to stop the practice, as education alone is not sufficient to end FGM/C [42, 59]. Similarly, Rawat [39], investigating FGM/C across six African countries including Sierra Leone found that education is significant in reducing FGM/C [39]. The current study's findings suggest that education is an important aspect in the fight against FGM/C and educated women may serve as anti-FGM/C advocates [50, 60].

Our findings highlight the ways in which wealth supports the discontinuation of FGM/C. Wealthier women and women who live in the western part of Sierra Leone are more likely to support the discontinuation of FGM/C. Concerning the association between wealth and support for the discontinuation of FGM/C, the findings corroborate previous studies that found a high socio-economic position helps protect against the practice of FGM/C [46, 61, 62]. Women with a higher level of education are more likely to be wealthy than those with no formal education [63]. Additionally, wealthy females with higher education tend to have less likelihood of FGM/C [56], which is why our finding was anticipated. The observed variation between the rich and poor may imply that women’s economic empowerment may reduce FGM/C [64].

Our study also found a geographical pattern regarding support for FGM/C discontinuation. Those residing in the country's western region were more likely to believe that FGM/C should be discontinued, while respondents from the northern region support its continuation. Several studies in Sierra Leone have found the western region residents have a high level of schooling, especially those in Freetown [65, 66]. Therefore, it is expected that women in the western region were more likely to support FGM/C's discontinuation. With high access to education in the region, women will gain more knowledge about the harmful effects of FGM/C, which may influence their perception of continuation. These findings give the impression that perception about the practice’s continuation or discontinuation varies significantly in Sierra Leone and possibly other countries [55]. The observed variations call for further research in the most FGM/C dominant region (northern) to improve understanding of the factors that promote the continuation of FGM/C and allow for appropriate interventions within communities [67].

The WHO stresses that education must be the centrepiece of multi-sectoral efforts aimed at stopping FGM/C [68]. Such measures must involve stakeholders at all levels (local, national and global) and varied sectors (health, economic, justice and education) [68]. These findings must be translated into action, as stating that FGM/C should be discontinued in itself is not sufficient to reduce its occurrence. Initial steps could involve strengthening the legal framework and motivating and awarding women who do not cut their daughters. Our study also revealed that despite the positive association between education and support for FGM/C discontinuation, more than half of the women sampled had no formal education. Almost 80% of them said that FGM/C should continue. This shows that the effectiveness of policies and strategies aimed at eliminating FGM/C will depend on non-formal education, which could target women with no formal education. This can be conducted through community sensitisation of parents, as they are the primary decision-makers regarding FGM/C practices.

## Strengths and limitations

One of the significant strengths of this study is that it is the first empirical study on education level and discontinuation of FGM/C in Sierra Leone, to the best of our knowledge. Second, the study is based on a nationally representative sample, a characteristic that makes it possible to generalise with ease. The inclusion of only significant variables reinforces the rigour of the models. The cross-sectional study design should be considered when interpreting the results of the study. Additionally, due to social factors such as religious affiliation and the community’s reaction to FGM/C, social desirability bias is possible.

## Conclusion

Education has been established as an important factor to help prevent the practice among women in Sierra Leone. This implies that FGM/C's discontinuation is a family and social affair, rather than an individual one. Many women will oppose FGM/C if they find themselves in families and communities that support their personal views. Therefore, while attitudinal change is a key step towards ending FGM/C, modification in social norms is required. There is a need to give critical attention to uneducated and poor women, especially those living in the southern part of Sierra Leone who may be at greater risk of FGM/C. Improving their education access is important, as well as linking girls and women to skills-based employment, particularly those who are already out-of-school, in unions, with daughters at home and those whose societies accept FGM/C. Enhanced access to education and employment may empower them to abandon this harmful practice. Research is also needed to assess whether formal education alone effectively ensures women’s support to discontinue FGM/C or if formal education combined with public health education would be more effective.

## Data Availability

Data used for the study is freely available to the public via https://dhsprogram.com/data/available-datasets.cfm.

## Abbreviations

AOR: Adjusted odds ratio
CI: Confidence interval
COR: Crude odds ratio
DHS: Demographic and Health Survey
FGM/C: Female genital mutilation/cutting
SDG: Sustainable development goals
SLDHS: Sierra Leone Demographic and Health Survey
VIF: Variance inflation factor
WHO: World Health Organisation
